# Novel cholesterol-high-density lipoprotein-glucose index is associated with 1-year recurrence of atrial fibrillation after catheter ablation

**DOI:** 10.3389/fcvm.2026.1982137

**Published:** 2026-10-29

**Authors:** Kamila Kamili, Suining Xu, Xiyang Li, Danni Wang, Pengjie Gao, Gang Tian, Haijian Xing

**Affiliations:** 1 Department of Cardiovascular Medicine, First Affiliated Hospital of Xi’an Jiaotong University, Xi’an, China; 2 Department of Outpatient Internal Medicine, Affiliated Children’s Hospital of Xi'an Jiaotong University, Xi'an, China

**Keywords:** atrial fibrillation recurrence, catheter ablation, cholesterol-high-density lipoprotein-glucose index, insulin resistance, triglyceride-glucose index

## Abstract

**Objective:**

Insulin resistance (IR) plays a pivotal role in the pathogenesis of atrial fibrillation (AF). The novel cholesterol-high-density lipoprotein-glucose (CHG) index, which integrates total cholesterol (TC), high-density lipoprotein cholesterol (HDL-C), and fasting blood glucose (FBG), has not been validated for predicting AF recurrence following radiofrequency catheter ablation (RFCA). This study aimed to evaluate the association between the CHG index and 1-year AF recurrence after RFCA, and to compare its performance with the triglyceride-glucose (TyG) index.

**Methods:**

A retrospective cohort of 813 AF patients who underwent first-time RFCA was enrolled. The primary outcome was any atrial tachyarrhythmia lasting ≥ 30 s after a 3-month blanking period. Cox regression, ROC curve, net reclassification improvement (NRI), and integrated discrimination improvement (IDI) were used to assess predictive performance.

**Results:**

During 1-year follow-up, 142 patients (17.5%) had recurrent atrial tachyarrhythmia, with significantly higher CHG levels in the recurrence group (*P* < 0.001). Each unit increase in the CHG index was associated with a 4.23-fold higher risk of recurrence (HR = 4.231, 95% CI: 3.406–5.257, *P* < 0.001). The restricted cubic spline curve shows a dose-response relationship between CHG and AF recurrence. The CHG index yielded an AUC of 0.765, superior to TyG (AUC = 0.673). Adding the CHG index to the traditional risk model significantly improved NRI (0.434, *P* < 0.001) and IDI (0.200, *P* < 0.001), with greater improvement than adding the TyG index. Subgroup analyses demonstrated consistent predictive effects across different populations.

**Conclusions:**

The CHG index is an independent risk marker for 1-year recurrence after AF ablation and demonstrates superior discriminative ability compared to the TyG index.

## 1 Introduction

Atrial fibrillation (AF) is the most common sustained cardiac arrhythmia, and radiofrequency catheter ablation has become a primary treatment option (1, 2). However, post-ablation AF recurrence remains a challenging clinical problem (3, 4). Therefore, identifying effective predictive markers to recognize high-risk patients and optimize post-procedural management is of great importance.

Recent studies have demonstrated that insulin resistance (IR) not only contributes to the pathogenesis of diabetes and metabolic syndrome but also increases AF susceptibility by promoting atrial electrical and structural remodeling (5, 6). A panel of IR surrogate biomarkers has been established based on routine laboratory tests. Among these, the triglyceride-glucose (TyG) index is the most well-validated metric (7), and accumulating clinical data have verified its predictive value for AF recurrence following RFCA (8–10). However, most of these indices focus primarily on triglycerides and glucose, with insufficient attention to cholesterol metabolism.

The cholesterol, high-density lipoprotein, and glucose (CHG) index is a newly proposed composite marker that incorporates total cholesterol (TC), high-density lipoprotein cholesterol (HDL-C), and fasting blood glucose (FBG) (11). Theoretically, it may provide a more comprehensive reflection of glucolipid metabolic disturbances. Nevertheless, no study has yet investigated the relationship between the CHG index and AF recurrence following ablation. Thus, this study aimed to analyze the association between the CHG index and 1-year recurrence after RFCA and further compare its predictive value with that of the TyG index.

## 2 Methods

### 2.1 Study population

This retrospective observational study consecutively enrolled patients undergoing their first radiofrequency ablation for AF at the Department of Cardiology, The First Affiliated Hospital of Xi'an Jiaotong University from January 2021 to January 2023. Exclusion criteria included: patients with advanced valvular heart disease or who underwent valve replacement or remodeling surgery; patients diagnosed with congenital heart disease, cardiomyopathy, uncontrolled hyperthyroidism, severe liver or renal dysfunction; and those with incomplete clinical data or loss to follow-up. A total of 813 patients were finally included (Figure 1). No patients died during the 12-month follow-up period.

**Figure 1:**
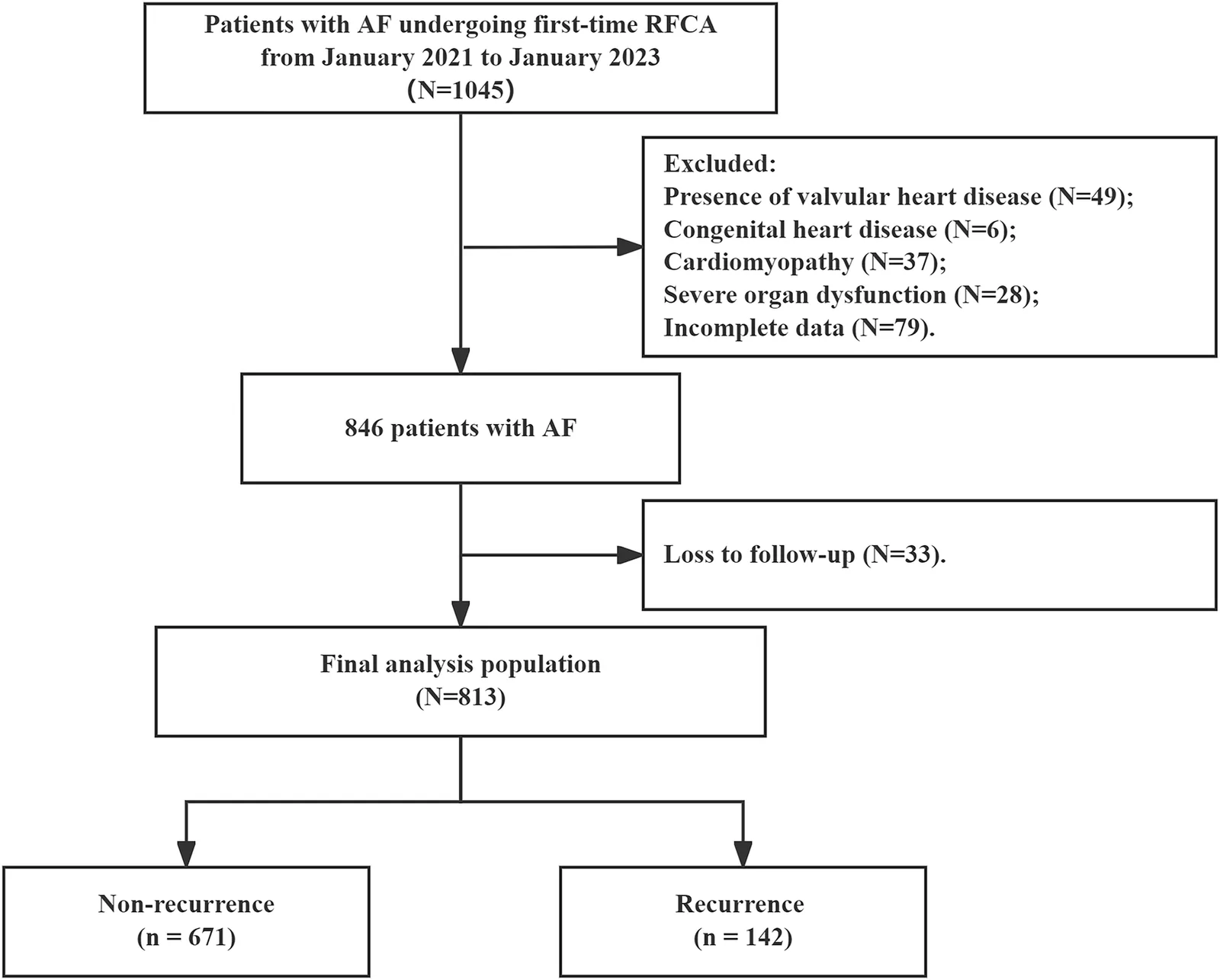
Flowchart of patient enrollment. AF, atrial fibrillation; RFCA, radiofrequency catheter ablation.

The study protocol was approved by the ethics committees of the First Affiliated Hospital of Xi'an Jiaotong University (No. XJTU1AF2018LSL-005), and the study adhered to the principles of the Declaration of Helsinki. All participants signed the written informed consent before they were included.

### 2.2 Data collection and definitions

Data were extracted from the hospital's electronic medical records, including demographic characteristics (age, sex), lifestyle factors (smoking, drinking), medical history (hypertension, diabetes mellitus, coronary artery disease, stroke, heart failure), AF type (paroxysmal/persistent), and medications at admission. Peripheral venous blood samples were taken after an overnight fast of more than 8 h. Transthoracic echocardiography was performed to evaluate the general anatomy of cardiac structures and cardiac function. The CHA2DS2-VASc score was recalculated based on the provided clinical information (12).

The CHG index was calculated using the formula: CHG index = Ln [TC (mg/dL) × FBG (mg/dL)/[2 × HDL-C (mg/dL)]] (11). Because TC, HDL-C and FBG were measured in mmol/L in our central laboratory, all values were converted to mg/dL before calculation using the following conversion factors: TC and HDL-C, 1 mmol/L = 38.67 mg/dL; FBG, 1 mmol/L = 18.0 mg/dL. The TyG index was calculated as TyG index = Ln [TG (mg/dL) × FBG (mg/dL)/2] (7), with TG converted from mmol/L to mg/dL using a factor of 88.57.

### 2.3 Ablation protocol and follow-up

All patients underwent circumferential pulmonary vein isolation guided by the CARTO three-dimensional mapping system (13). Additional linear ablation was performed when indicated, particularly in patients with persistent AF (14). The first three months after ablation were defined as the blanking period, during which all patients received antiarrhythmic drugs. Follow-up visits were scheduled every 3 months for 1 year, with each visit including a 12-lead electrocardiogram and 24 h Holter monitoring. The primary endpoint was the first documented recurrence of any atrial tachyarrhythmia (including atrial fibrillation, atrial flutter, or atrial tachycardia) lasting ≥ 30 s after the 3-month blanking period, as confirmed by 12-lead ECG or 24 hour Holter monitoring (15).

### 2.4 Statistical analysis

Continuous variables were expressed as median with interquartile range, and categorical variables as counts (percentages). Group comparisons were performed using the Mann–Whitney *U* test or chi-square test. Multivariable Cox proportional hazards models were used to assess the correlation between the CHG index and AF recurrence. Receiver operating characteristic (ROC) curve analysis was used to evaluate the discriminative performance of the CHG index for 1-year recurrence. The dose-response relationship between CHG index and AF recurrence was examined using restricted cubic spline (RCS) analysis. For assessment of incremental predictive value, the pre-specified basic model included age, sex, smoking and drinking status, diabetes mellitus, resting heart rate, body mass index, left atrial diameter, CHA_2_DS_2_-VASc score, and AF type. These covariates were selected *a priori* based on established predictors of post-ablation AF recurrence reported in prior literature and their inclusion was further confirmed by univariable Cox regression screening (all with *P* < 0.10). Survival analyses used the date of the index ablation as the time origin; because arrhythmias occurring during the 3-month blanking period were not counted as events, patients were considered at risk for the endpoint from the end of the blanking period and were followed until the first documented endpoint event, the last available follow-up, or 12 months after ablation, whichever came first. All metrics were evaluated for the 1-year post-ablation prediction horizon, matching the primary endpoint definition. Continuous (category-free) net reclassification improvement (NRI) and integrated discrimination improvement (IDI) were calculated using the method of Pencina et al. (16) with inverse probability of censoring weighting (IPCW) to properly account for censored observations during follow-up, as implemented in the R nricens and survIDINRI packages. Model discrimination was assessed using Harrell's C-index. Calibration was evaluated graphically with calibration curves (1000-bootstrap bias correction). Internal validation was performed using 1000-resample bootstrapping to estimate optimism-corrected C-index and calibration performance for both the baseline model and the CHG-extended model. All NRI/IDI and discrimination metrics were computed for the 12-month risk of AF recurrence after catheter ablation, matching the primary endpoint definition. Subgroup analyses were stratified by sex, age (≤60 or >60 years), AF type, hypertension, and diabetes mellitus (DM). All tests were two-sided, with *P* < 0.05 considered statistically significant. Statistical analyses were performed using SPSS 26.0 and R 4.0.3.

## 3 Results

### 3.1 Baseline patient characteristics

A total of 813 AF patients were included, of whom 142 (17.5%) experienced recurrence during the 1-year follow-up period. As shown in Table 1, compared with the non-recurrence group, patients with recurrence had higher proportions of diabetes mellitus and persistent AF (both *P* < 0.001). The recurrence group had a significantly higher CHG index and TyG index (all *P* < 0.05). Additionally, body mass index (BMI), heart rate (HR), red blood cell count (RBC), high-sensitivity C-reactive protein (hs-CRP), uric acid (UA), triglycerides (TG), low-density lipoprotein cholesterol (LDL-C), N-terminal pro-brain natriuretic peptide (NT-proBNP), cardiac troponin T (cTnT), left ventricular end-systolic diameter (LVESD), right ventricular internal diameter (RVID), and left atrial diameter (LAD) were found to be higher in the recurrence group. But no significant differences were observed between the two groups in terms of age, sex, smoking, or alcohol consumption, history of coronary heart disease, stroke, and heart failure. The baseline characteristics of patients stratified by CHG index quartiles are presented in Supplementary Table S1, showing that the proportion of atrial fibrillation recurrence increased progressively with higher CHG index levels.

**Table 1:** Baseline characteristics of participants by recurrence following ablation.

Variables	Overall (*n* = 813)	Non-recurrence (*n* = 671)	Recurrence (*n* = 142)	*P* value
Persistent AF, *n* (%)	303 (37.27)	226 (33.68)	77 (54.23)	<0.001
Age, years	64.0 [56.0, 69.0]	64.0 [56.0, 69.0]	64.0 [58.0, 71.0]	0.268
Male, *n* (%)	506 (62.24)	411 (61.25)	95 (66.90)	0.207
BMI, kg/m^2^	24.91[22.96, 26.95]	24.79[22.86, 26.78]	25.65 [23.51, 27.72]	0.009
CHA_2_DS_2_-VASc score	2.0 [1.0, 3.0]	2.0 [1.0, 3.0]	2.0 [1.0, 3.0]	0.061
HAS-BLED score	1.0 [0.0, 2.0]	1.0 [0.0, 2.0]	1.0 [0.0, 2.0]	0.117
SBP, mmHg	123.0 [112.0, 137.0]	123.0 [112.0, 137.0]	126.0 [110.0, 136.0]	0.571
DBP, mmHg	79.0 [72.0, 87.0]	79.0 [72.0, 86.0]	80.0 [73.0, 88.0]	0.149
HR, BPM	74.0 [65.0, 83.0]	74.0 [65.0, 82.0]	78.0 [66.0, 87.0]	0.024
Current smoking, *n* (%)	267 (32.84)	220 (32.79)	47 (33.10)	0.943
Current drinking, *n* (%)	98 (12.05)	86 (12.82)	12 (8.45)	0.147
Hypertension, *n* (%)	435 (53.51)	358 (53.35)	77 (54.23)	0.85
Diabetes mellitus, *n* (%)	140 (17.22)	96 (14.31)	44 (30.99)	<0.001
CHD, *n* (%)	149 (18.33)	116 (17.29)	33 (23.24)	0.096
Heart failure, *n* (%)	30 (3.69)	25 (3.73)	5 (3.52)	0.906
Prior stroke, *n* (%)	111 (13.65)	88 (13.12)	23 (16.20)	0.331
Anticoagulants, *n* (%)	302 (37.15)	238 (35.47)	64 (45.07)	0.091
ACEI/ARB, *n* (%)	241 (29.64)	207 (30.85)	34 (23.94)	0.102
Beta-blockers, *n* (%)	367 (45.14)	308 (45.90)	59 (41.55)	0.344
Statins, *n* (%)	247 (30.38)	202 (30.10)	45 (31.69)	0.709
OADs, *n* (%)	93 (11.48)	60 (8.97)	33 (23.40)	<0.001
RBC, ×10^12^/L	4.54 [4.26, 4.92]	4.53 [4.24, 4.89]	4.65 [4.39, 5.02]	0.019
HGB, g/L	143.00 [131.00, 154.00]	142.00 [131.00, 154.00]	144.00 [132.00, 154.00]	0.357
PLT, 109/L	188.00 [150.00, 224.00]	190.00 [152.00, 226.00]	177.00 [146.00, 218.00]	0.07
WBC, 10^9^/L	5.79 [4.77, 6.91]	5.76 [4.75, 6.87]	5.94 [5.09, 7.35]	0.061
hs-CRP, mg/L	2.17 [0.80, 5.08]	2.07 [0.77, 4.88]	3.00 [0.92, 6.29]	0.03
ALB, g/L	39.30 [37.00, 41.80]	39.40 [37.00, 41.80]	39.20 [37.00, 41.70]	0.849
BUN, mmol/L	6.36 [5.28, 7.58]	6.37 [5.27, 7.59]	6.25 [5.38, 7.54]	0.931
Scr, μmol/L	64.00 [53.00, 76.00]	63.00 [53.00, 75.00]	67.00 [54.00, 78.00]	0.054
FBG, mmol/L	5.64 [4.90, 7.02]	5.49 [4.82, 6.66]	6.97 [5.41, 9.23]	<0.001
UA, μmol/L	319.00 [264.00, 376.00]	311.00 [259.00, 370.00]	353.00 [285.00, 415.00]	<0.001
TC, mmol/L	3.48 [2.92, 4.07]	3.44 [2.92, 4.04]	3.69 [3.02, 4.13]	0.062
TG, mmol/L	1.08 [0.80, 1.54]	1.05 [0.79, 1.51]	1.18 [0.93, 1.70]	0.006
HDL-C, mmol/L	0.99 [0.85, 1.15]	1.00 [0.88, 1.16]	0.92 [0.77, 1.09]	<0.001
LDL-C, mmol/L	1.95 [1.41, 2.49]	1.90 [1.38, 2.47]	2.11 [1.57, 2.58]	0.013
NT-proBNP, pg/mL	431.40 [140.10, 938.60]	382.70 [121.00, 883.90]	742.30 [312.40, 1,116.00]	<0.001
cTnT, ng/mL	0.007 [0.005, 0.011]	0.007 [0.005, 0.011]	0.009 [0.006, 0.015]	<0.001
LVEDD, mm	48.0 [46.0, 52.0]	48.0 [46.0, 52.0]	49.0[47.0, 52.0]	0.088
LVESD, mm	31.0 [28.0, 34.0]	30.0 [28.0, 34.0]	32.0[29.0, 36.0]	<0.001
IVSD, mm	8.0 [8.0, 9.0]	8.0 [8.0, 9.0]	8.0[8.0, 9.0]	0.458
RVID, mm	16.0 [16.0, 18.0]	16.0 [15.0, 18.0]	17.0 [16.0, 19.0]	<0.001
LVEF, %	67.0 [62.0, 70.0]	67.0 [62.0, 70.0]	65.0 [60.0, 69.0]	0.009
LAD, mm	36.0 [32.0, 40.0]	35.0 [31.0, 39.0]	41.0 [36.0, 46.0]	<0.001
TyG index	8.58 [8.17, 9.00]	8.47 [8.08, 8.91]	8.92 [8.54, 9.24]	<0.001
CHG index	5.22 [4.96, 5.48]	5.17 [4.91, 5.42]	5.56 [5.27, 5.92]	<0.001

AF, atrial fibrillation; ACEI/ARB, angiotensin-converting enzyme inhibitors/angiotensin II receptor antagonists; ALB, serum albumin; BMI, body mass index; BUN, blood urea nitrogen; CHD, coronary heart disease; CHG, cholesterol, high-density lipoprotein, and glucose index; cTnT, cardiac troponin T; DBP, diastolic blood pressure; FBG, fasting blood glucose; HDL-C, high-density lipoprotein cholesterol; HR, heart rate; hs-CRP, high-sensitivity C-reactive protein; IVSD, interventricular septal depth; LAD, left atrial diameter; LDL-C, low-density lipoprotein cholesterol; LVEDD, left ventricular end-diastolic diameter; LVEF, left ventricular ejection fraction; LVESD, left ventricular end-systolic diameter; NT-proBNP, N-terminal pro-brain natriuretic peptide; OADs, oral antidiabetic agents; PLT, platelet; RBC, red blood cells; RVID, right ventricular internal diameter; SBP, systolic blood pressure; Scr, serum creatinine; TC, total cholesterol; TG, triglyceride; TyG, triglyceride glucose index; WBC, white blood cells.

### 3.2 Association between the CHG index and AF recurrence

As shown in Table 2, univariate Cox regression revealed a significant association between the CHG index and AF recurrence (HR = 3.424, 95% CI: 2.817–4.161, *P* < 0.001). After adjusting for age and sex in Model 1, the HR was 3.353 (95% CI: 2.759–4.075, *P* < 0.001). The fully adjusted model (Model 2) further confirmed the association between elevated CHG and AF recurrence (HR = 4.231, 95% CI: 3.406–5.257, *P* < 0.001). When the CHG index was categorized into quartiles, using the lowest quartile (Q1, ≤4.95) as the reference, a graded increase in AF recurrence risk was observed across quartiles. The crude hazard ratios were 2.506 (95% CI: 1.039–6.044, *P* = 0.041) for Q2, 5.826 (95% CI: 2.614–12.986, *P* < 0.001) for Q3 and 13.907 (95% CI: 6.412–30.160, *P* < 0.001) for Q4, and the graded association persisted in the fully adjusted mode.

**Table 2:** Hazard ratios for associations between the CHG and TyG indexes and AF recurrence.

	*N* (%)	Crude			Model 1			Model 2		
Variables		HR	95%CI	*P*-value	HR	95%CI	*P*-value	HR	95%CI	*P*-value
CHG	142 (17.5)	3.424	2.817, 4.161	<0.001	3.353	2.759, 4.075	<0.001	4.231	3.406, 5.257	<0.001
Q1	7 (3.5)	ref.	ref.	ref.	ref.	ref.	ref.	ref.	ref.	ref.
Q2	17 (8.6)	2.506	1.039, 6.044	0.041	2.554	1.058, 6.165	0.037	2.440	1.008, 5.907	0.048
Q3	41 (19.2)	5.826	2.614, 12.986	<0.001	6.105	2.735, 13.628	<0.001	6.630	2.956, 14.872	<0.001
Q4	77 (37.9)	13.907	6.412, 30.160	<0.001	14.713	6.772, 31.963	<0.001	18.296	8.282, 40.418	<0.001
*P* for trend				<0.001			<0.001			<0.001
TyG	142 (17.5)	2.434	1.86, 3.185	0.001	2.567	1.957, 3.366	<0.001	3.655	2.661, 5.02	<0.001
Q1	11 (5.4)	ref.	ref.	ref.	ref.	ref.	ref.	ref.	ref.	ref.
Q2	30 (14.8)	2.836	1.421, 5.659	0.003	2.990	1.497, 5.973	0.002	2.829	1.408, 5.683	0.003
Q3	41 (20.2)	3.996	2.054, 7.774	<0.001	4.303	2.207, 8.388	<0.001	3.965	2.014, 7.804	<0.001
Q4	60 (29.6)	6.424	3.377, 12.222	<0.001	7.159	3.746, 13.681	<0.001	10.955	5.589, 21.475	<0.001
*P* for trend				<0.001			<0.001			<0.001

Model 1 was adjusted for age and sex; Model 2 was further adjusted for smoking and drinking status, diabetes mellitus, heart rate, body mass index, left atrial diameter, CHA_2_DS_2_-VASc score, and atrial fibrillation type.

Quartile of CHG index: Q1: CHG ≤ 4.953, Q2: 4.95 < CHG ≤ 5.22, Q3: 5.22 < CHG ≤ 5.49, Q4: CHG ≥ 5.49.

Quartile of TyG index: Q1: TyG ≤ 8.164, Q2: 8.164 < TyG  ≤ 8.575, Q3: 8.575 < TyG ≤ 9.004, Q4: TyG ≥ 9.004.

CHG, cholesterol, high-density lipoprotein and glucose index; CI, confidence interval; HR, hazard ratio; TyG, triglyceride glucose index.

For the TyG index, after fully adjusting for age, sex, smoking and drinking status, DM, HR, BMI, LAD, CHA_2_DS_2_-VASc score, and AF type, each 1-unit increase was associated with a 265.5% % increase in AF recurrence risk (HR = 3.655, 95% CI: 2.661–5.020, *P* < 0.001). When the TyG index was analyzed as a categorical variable, patients in the highest quartile exhibited a 10.955-fold higher risk of atrial fibrillation recurrence compared with those in the first quartile (fully adjusted HR = 10.955, 95% CI:5.589–21.475, *P* < 0.001; *P* for trend < 0.001).

To examine the dose-response relationship between the CHG index and AF recurrence, an RCS analysis was conducted (Figure 2). Both indices were significantly associated with recurrence overall (*P* for overall < 0.001 for both). No significant nonlinearity was detected for the TyG index (*P* for nonlinearity = 0.707), indicating an essentially linear association, whereas the CHG index showed a significant nonlinear association (*P* for nonlinearity < 0.001).

**Figure 2:**
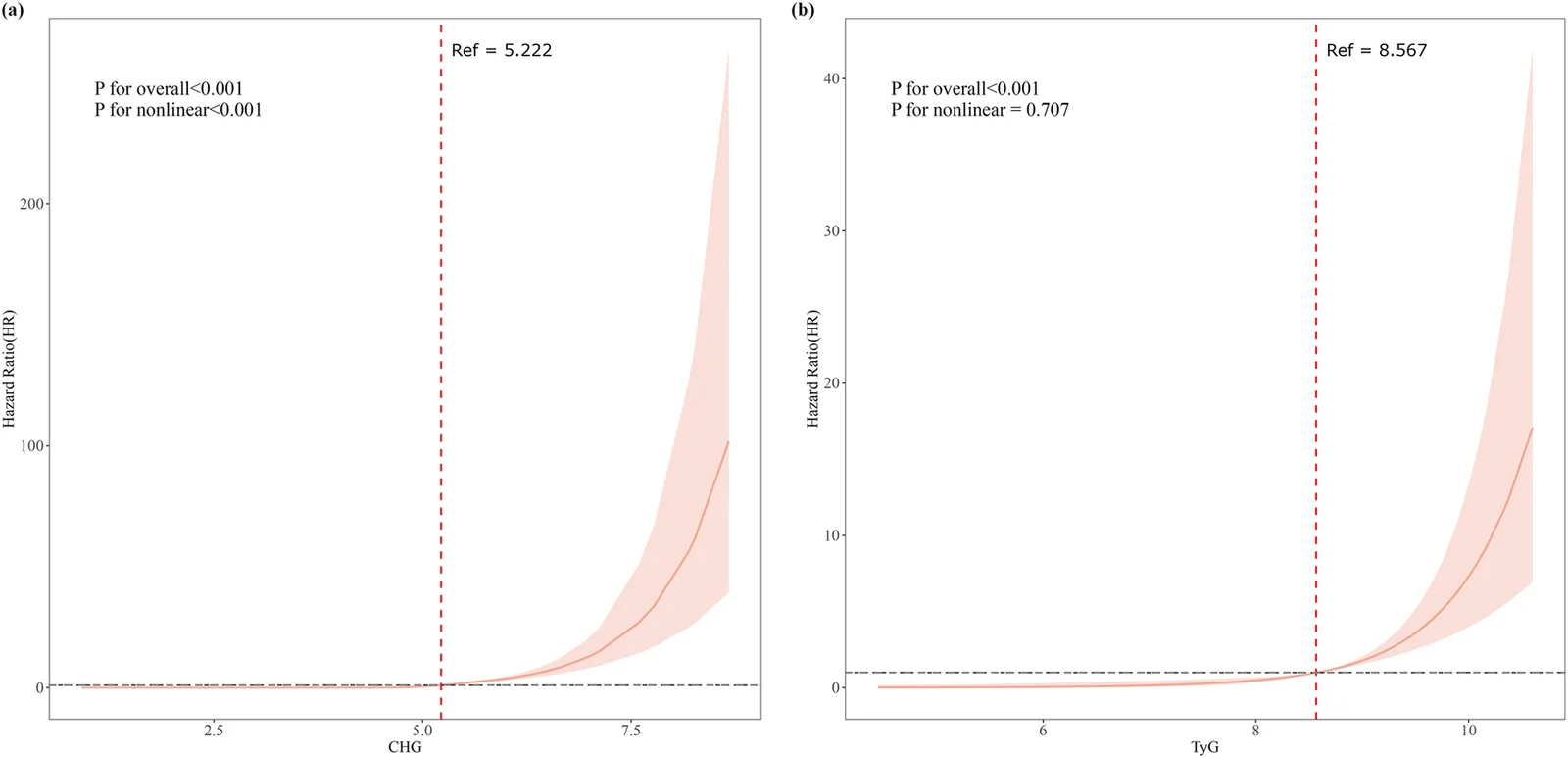
Restricted cubic spline models analyzed the relationship between cholesterol, high-density lipoprotein, glucose index **(a)** and triglyceride–glucose index **(b)**, and 1-year atrial fibrillation recurrence.

### 3.3 Kaplan-meier survival analysis

Patients were divided into four groups according to quartiles of the CHG index. As shown in Figure 3. Kaplan–Meier survival analysis revealed a significant difference in recurrence-free survival time among the four groups (log-rank *χ*^2^ = 98.62, *P* < 0.001). As the CHG index increased, recurrence-free survival time progressively shortened, with the earliest recurrences observed in the highest quartile group (Q4). Similarly, when patients were stratified by quartiles of the TyG index, Kaplan–Meier analysis also demonstrated a significant difference in recurrence-free survival time across the four groups (log-rank *χ*^2^ = 50.88, *P* < 0.001).

**Figure 3:**
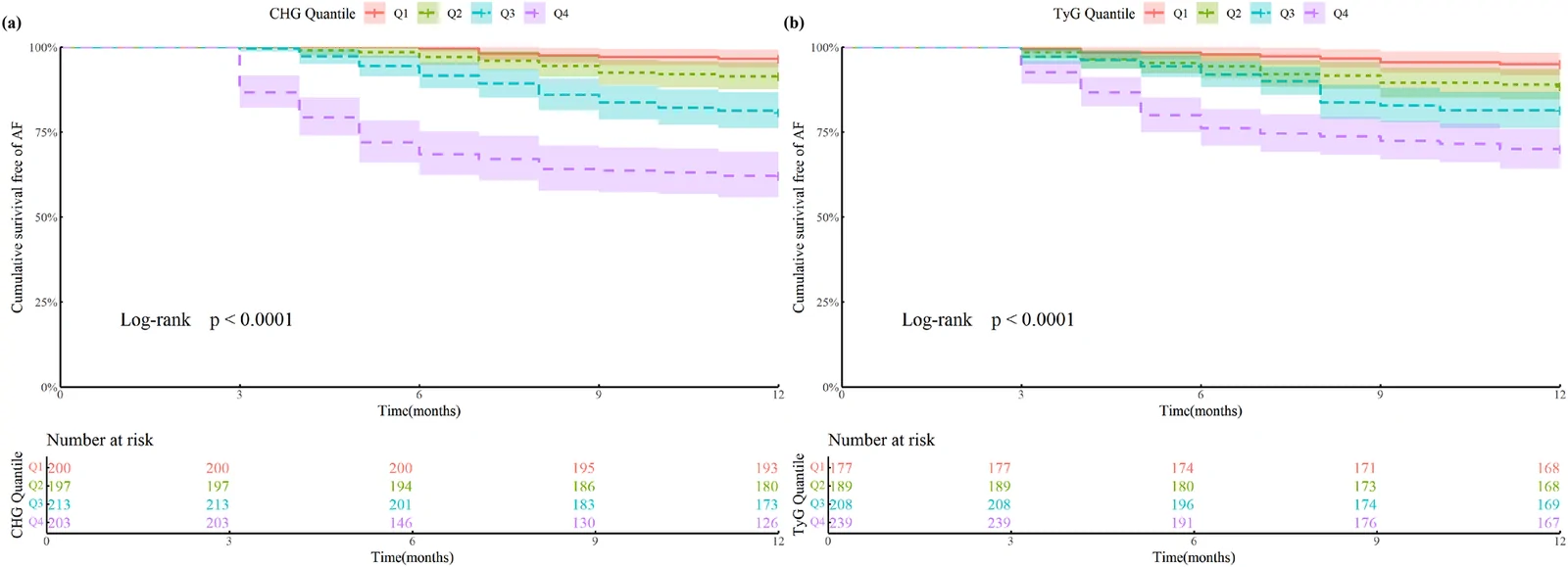
Kaplan–meier curves for cumulative atrial fibrillation-free survival stratified by cholesterol-high-density lipoprotein-glucose (CHG) index **(a)** and triglyceride-glucose (TyG) index **(b)**.

### 3.4 Comparison of the discriminative performance of the CHG index and the TyG index for AF recurrence

ROC curve analysis showed that the CHG index had an area under the curve of 0.765 (95% CI: 0.727–0.803) for discriminating AF recurrence, with an optimal cutoff value of 5.23, corresponding to a sensitivity of 81.7% and specificity of 58.1% (Supplementary Figure S1; Supplementary Table S2). For comparison, the TyG index had an AUC of 0.673 (95% CI: 0.633–0.713). The DeLong test showed that the CHG index exerts superior independent discriminatory power compared with the TyG index (*P* < 0.001).

As shown in Table 3, adding the CHG index to the baseline model significantly improved reclassification, with an NRI of 0.434 (95% CI: 0.223–0.603, *P* < 0.001) and an IDI of 0.200 (95% CI: 0.127–0.250, *P* < 0.001). Adding the TyG index yielded an NRI of 0.263 (95% CI: 0.029–0.390, *P* < 0.001) and an IDI of 0.064 (95% CI: 0.007–0.122, *P* = 0.012). When compared with the base model incorporating the TyG index, adding the CHG index to the base model demonstrates superior incremental discriminative value for atrial fibrillation recurrence (NRI = 0.365, IDI = 0.135, both *P* < 0.05).

**Table 3:** Incremental predictive value of indicators derived from NRI and IDI.

Indicator	NRI (95%CI)	*P*	IDI (95%CI)	*P*
Basic model	Ref.	Ref.	Ref.	Ref.
Basic model + TyG	0.263 (0.029, 0.390)	<0.001	0.064 (0.007, 0.122)	0.012
Basic model + CHG	0.434 (0.223, 0.603)	<0.001	0.200 (0.127, 0.250)	<0.001
	0.365 (0.076, 0.492)^a^	<0.001^a^	0.135 (0.058, 0.201)^a^	0.012^a^

Basic model, adjusted for age, sex, smoking and drinking status, diabetes mellitus, heart rate, body mass index, left atrial diameter, CHA_2_DS_2_-VASc score and atrial fibrillation type.

CHG, cholesterol, high-density lipoprotein and glucose index; CI, confidence interval; IDI, integrated discrimination improvement; NRI, reclassification improvement; TyG, triglyceride glucose index.

a Basic model + CHG index compared to Basic model + Tyg index.

Regarding discrimination, the baseline clinical model achieved an apparent Harrell's C-index of 0.628 for 12-month post-ablation AF recurrence, which increased to 0.753 after adding the CHG index. After 1000-bootstrap optimism correction, the C-indices were 0.600 (optimism = 0.028) and 0.735 (optimism = 0.017), respectively, indicating that the improvement was robust rather than attributable to overfitting (Supplementary Table S3). Calibration curves with bootstrap bias correction (Supplementary Figure S2) showed slight risk overestimation at higher predicted probabilities for the baseline model, whereas the CHG-extended model aligned closely with observed event rates across the risk range.

To assess whether CHG adds prognostic information beyond its components, we compared models containing FBG, HDL-C, or TC/HDL-C individually against the CHG model. FBG alone improved reclassification (NRI: 0.336, 95% CI: 0.140–0.542, *P* = 0.010; IDI: 0.082, 95% CI: 0.029–0.125, *P* < 0.001) but remained inferior to CHG (NRI: 0.241, 95% CI: 0.026–0.441, *P* = 0.030; IDI: 0.118, 95% CI: 0.060–0.198, *P* < 0.001). HDL-C alone did not significantly improve reclassification and was markedly outperformed by CHG (NRI: 0.507, 95% CI: 0.181–0.665; IDI: 0.183, 95% CI: 0.105–0.235; both *P* < 0.001). TC/HDL-C alone yielded modest improvement (NRI: 0.264, 95% CI: 0.039–0.375, *P* = 0.030; IDI: 0.057, 95% CI: 0.019–0.102, *P* < 0.001) but was also surpassed by CHG (NRI: 0.441, 95% CI: 0.207–0.648; IDI: 0.143, 95% CI: 0.079–0.193; both *P* < 0.001). These component-wise comparisons indicate that the composite CHG index provides synergistic prognostic information beyond any single constituent (Supplementary Table S4).

### 3.5 Subgroup analysis

Subgroup analyses by AF type, sex, hypertension, DM, and age are presented in Supplementary Figure S3. In all subgroups, the CHG index remained significantly associated with an increased risk of 1-year atrial fibrillation recurrence. These results fully confirmed the robustness of the study findings.

## 4 Discussion

The present study suggests that the CHG index may be a stronger predictor of AF recurrence following catheter ablation compared with the traditional TyG index. In a cohort of 813 patients, we demonstrated that elevated CHG levels are independently associated with a significantly increased risk of AF recurrence, with a hazard ratio of 4.231 (95% CI: 3.406–5.257) in the fully adjusted model. Crucially, the CHG index exhibited superior discriminative performance, with an AUC of 0.765 compared to 0.673 for the TyG index, and provided significant incremental value in risk reclassification. These findings are consistent with the hypothesis that comprehensive glucolipid metabolic dysregulation, rather than insulin resistance alone, may contribute to the structural and electrical remodeling that predisposes patients to post-ablation AF recurrence.

The superior predictive value of the CHG index likely stems from its ability to capture a broader spectrum of metabolic pathophysiology relevant to atrial remodeling. While the TyG index is a well-established surrogate for insulin resistance and hepatic glucose production, it primarily reflects triglyceride-rich lipoprotein dynamics (8, 17). In contrast, the CHG index integrates total cholesterol and HDL-C, thereby accounting for the atherogenic potential of non-HDL cholesterol and the protective, anti-inflammatory properties of HDL-C (18). Recent reviews highlight that metabolic syndrome components, including dyslipidemia, contribute to atrial remodeling through lipotoxicity, mitochondrial dysfunction, and altered energy substrate utilization (19, 20). The inclusion of cholesterol metrics in the CHG index allows for a more holistic assessment of systemic metabolic stress, which is increasingly recognized as a key driver of atrial cardiomyopathy (17, 21).

A plausible mechanism linking the CHG index to AF recurrence involves cholesterol metabolism in atrial inflammation and fibrosis. Dysregulated cholesterol levels promote oxidative stress via NADPH oxidase activation and stimulate inflammatory signaling pathways, such as the NLRP3 inflammasome, leading to the release of pro-inflammatory cytokines like IL-1β and TNF-α (22, 23). These inflammatory mediators recruit macrophages to the atrial myocardium, driving fibrotic signaling and interstitial fibrosis, which creates the structural substrate for reentrant arrhythmias (23, 24). Furthermore, HDL-C exerts cardioprotective effects through reverse cholesterol transport and antioxidant properties, but its functionality can be impaired in states of chronic inflammation, a hallmark of AF (23, 25). By capturing both quantitative lipid abnormalities and potential functional impairments, the CHG index may serve as a proxy for the pro-inflammatory and pro-fibrotic milieu that has been associated with poorer outcomes after catheter ablation (21, 26).

Our finding of a significant nonlinear relationship between the CHG index and AF recurrence, as demonstrated by restricted cubic spline analysis, further supports the complex nature of this metabolic-arrhythmic link. This nonlinear dose-response relationship suggests that there may be threshold effects or synergistic interactions between glucose and cholesterol metabolism that exacerbate atrial remodeling at higher levels of metabolic dysfunction (18, 20). In contrast, while the TyG index also showed a significant association with recurrence, its linear or less complex relationship may not fully capture the multifaceted metabolic insults driving AF progression (8, 27). The superior NRI and IDI observed when adding the CHG index to baseline models support its potential utility in refining risk stratification beyond traditional clinical factors and even other metabolic markers (8, 28).

Consistent findings were observed across subgroups, in which the CHG index remained a significant predictor of AF recurrence in patients with diabetes mellitus, hypertension, and different AF types (19, 29). This consistency suggests that glucolipid metabolic health may be relevant to AF management across comorbid conditions. Given that AF is linked to systemic metabolic dysregulation, the CHG index may offer a practical and cost-effective means of identifying high-risk patients who could be candidates for closer metabolic surveillance; whether such surveillance or treatment alters outcomes remains to be determined (20, 21).

Clinically, the CHG index integrates FBG, TC, and HDL-C, reflecting comprehensive glucolipid metabolic dysregulation. Because metabolic status is potentially modifiable, whether its modification influences recurrence after ablation is of direct clinical relevance. Recent evidence from the China-AF Registry shows that sodium-glucose cotransporter 2 inhibitor (SGLT2i) therapy is associated with lower AF recurrence after catheter ablation in patients with chronic kidney disease (CKD), metabolic syndrome, or diabetes (HRs: 0.56–0.63) (30–32). However, in the DARE-AF randomized trial (15), three months of dapagliflozin did not reduce AF burden (7.5% vs. 8.1%; *P* = 0.48) or early recurrence (HR: 1.11, 95% CI: 0.66–1.86; *P* = 0.70) in patients without diabetes, heart failure or chronic kidney disease. Taken together, these data suggest that the benefit of metabolic therapy may depend on the presence of a demonstrable metabolic abnormality, the population in which an index such as CHG would be expected to be informative. Patients with elevated CHG indices may warrant closer surveillance and optimisation of metabolic risk factors, which is consistent with current guideline-recommended cardiovascular risk management; whether risk stratification by the CHG index, followed by intensified modification of metabolic status, can reduce recurrence after ablation remains to be tested in prospective randomized trials.

This study has several limitations. First, it was a single-center retrospective study, which may introduce selection bias and unmeasured confounding factors. Second, the follow-up duration was limited to one year, so the long-term predictive value of the CHG index requires further validation. Third, although all patients underwent circumferential pulmonary vein isolation and received antiarrhythmic drugs during the blanking period, other treatment and procedural details were not systematically captured. These unmeasured factors may represent a source of residual confounding, and some recurrences may have been suppressed by ongoing antiarrhythmic therapy. Fourth, follow-up relied on scheduled 12-lead ECG and 24 h Holter monitoring, which may miss asymptomatic or brief recurrences; the observed recurrence rate is therefore likely to be an underestimate, and continuous monitoring might identify additional episodes and alter the estimated performance of the CHG index. Finally, the optimal CHG cutoff value of 5.23 derived from the ROC Youden index in the present cohort was internally validated but requires external validation in independent multicenter cohorts before a clinically generalizable threshold can be established.

## 5 Conclusion

In conclusion, the CHG index is an independent risk marker of 1-year AF recurrence after RFCA. It exhibits superior discriminative performance and incremental clinical value compared with the conventional TyG index.

## Data Availability

The raw data supporting the conclusions of this article will be made available by the authors, without undue reservation.
