# Supplementary material for: Novel cholesterol-high-density lipoprotein-glucose index is associated with 1-year recurrence of atrial fibrillation after catheter ablation
Source: Front Cardiovasc Med. 2026 Oct 29;13:1982137. doi: 10.3389/fcvm.2026.1982137 (PMC13754664; doi:10.3389/fcvm.2026.1982137)
Supplement: Supplementary file 1 [file Datasheet1.docx]

**Table S1 Baseline characteristics of participants by CHG index levels.**

| **Variables** | **Overall**  **(n=813)** | **Q1**  **(n=200)** | **Q2**  **(n=197)** | **Q3**  **(n=213)** | **Q4**  **(n=203)** | **P value** |
| --- | --- | --- | --- | --- | --- | --- |
| Persistent AF, n (%) | 303(37.27) | 83(41.50) | 80(40.61) | 70(32.86) | 70(34.48) | 0.178 |
| Age, years | 64.0[56.0,69.0] | 65.0[59.0,70.0] | 63.0[56.0,69.0] | 63.0[55.0,68.0] | 63.0[56.0,70.0] | 0.042 |
| Male, n (%) | 506(62.24) | 114(57.00) | 129(65.48) | 136(63.85) | 127(62.56) | 0.326 |
| BMI, kg/m^2^ | 24.91[22.96,26.95] | 24.22[22.04,26.79] | 24.64[22.49,26.58] | 25.39[23.51,27.10] | 25.15[23.51,27.14] | 0.005 |
| CHA2DS2-VASc score | 2.0[1.0,3.0] | 2.0[1.0,3.0] | 2.0[1.0,3.0] | 2.0[1.0,3.0] | 2.0[1.0,3.0] | 0.109 |
| Hasbled score | 1.0[0.0,2.0] | 1.0[0.0,2.0] | 1.0[0.0,2.0] | 1.0[0.0,2.0] | 1.0[0.0,2.0] | 0.21 |
| SBP, mmHg | 123.0[112.0,137.0] | 123.0[110.0,139.0] | 121.0[110.0,136.0] | 123.0[110.0,136.0] | 126.0[113.0,136.0] | 0.484 |
| DBP, mmHg | 79.0[72.0,87.0] | 77.0[71.0,85.0] | 79.000[73.0,87.0] | 79.000[71.0,87.0] | 81.0[73.0,87.0] | 0.123 |
| HR, bpm | 74.0[65.0,83.0] | 74.0[64.0,83.0] | 73.0[64.0,81.0] | 73.0[65.0,85.0] | 76.0[66.0,86.0] | 0.087 |
| Current smoking, n (%) | 267(32.84) | 64(32.00) | 65(33.00) | 75(35.21) | 63(31.03) | 0.823 |
| Current drinking, n (%) | 98(12.05) | 19(9.50) | 36(18.27) | 23(10.80) | 20(9.85) | 0.022 |
| Hypertension, n (%) | 435(53.51) | 109(54.50) | 101(51.27) | 110(51.64) | 115(56.65) | 0.664 |
| Diabetes mellitus, n (%) | 140(17.22) | 13(6.50) | 24(12.18) | 32(15.02) | 71(34.98) | <0.001 |
| CHD, n (%) | 149(18.33) | 46(23.00) | 29(14.72) | 35(16.43) | 39(19.21) | 0.155 |
| Heart failure, n (%) | 30(3.69) | 7(3.50) | 7(3.55) | 9(4.23) | 7(3.45) | 0.972 |
| Prior stroke, n (%) | 111(13.65) | 27(13.50) | 31(15.74) | 28(13.15) | 25(12.32) | 0.781 |
| NOAC, n (%) | 302(37.15) | 79(39.50) | 78(39.59) | 73(34.27) | 72(35.47) | 0.52 |
| ACEI/ARB, n (%) | 241(29.64) | 64(32.00) | 63(31.98) | 61(28.64) | 53(26.11) | 0.5 |
| Betablockers, n (%) | 367(45.14) | 89(44.50) | 94(47.72) | 94(44.13) | 90(44.34) | 0.873 |
| CCBs, n (%) | 200(24.63) | 53(26.50) | 42(21.43) | 50(23.47) | 55(27.09) | 0.517 |
| Statins, n (%) | 247(30.38) | 78(39.01) | 56(28.43) | 46(21.60) | 67(33.01) | 0.001 |
| Anticoagulants, n (%) | 180(22.14) | 54(27.00) | 44(22.34) | 33(15.49) | 49(24.14) | 0.075 |
| OAD, n (%) | 93(11.48) | 8(4.00) | 15(7.65) | 24(11.32) | 46(22.77) | <0.001 |
| RBC, ×10^12^/L | 4.54[4.26,4.92] | 4.44[4.16,4.85] | 4.54[4.26,4.98] | 4.65[4.36,4.98] | 4.58[4.30,4.85] | 0.001 |
| HGB, g/L | 143.00[131.00,154.00] | 139.00[129.00,151.00] | 143.00[131.00,155.00] | 145.00[133.00,156.00] | 143.00[132.00,154.00] | 0.023 |
| PLT, 10^9^/L | 188.00[150.00,224.00] | 177.00[142.00,214.00] | 193.00[152.00,223.00] | 187.00[149.00,232.00] | 197.00[156.00,234.00] | 0.009 |
| WBC, 10^9^/L | 5.79[4.77,6.91] | 5.54[4.60,6.58] | 5.69[4.70,6.68] | 5.89[4.96,7.15] | 6.13[5.06,7.47] | 0.003 |
| hs-CRP, mg/L | 2.17[0.80,5.08] | 2.63[0.83,5.76] | 1.59[0.65,4.29] | 2.41[0.91,5.08] | 1.96[0.75,4.74] | 0.056 |
| ALB, g/L | 39.30[37.00,41.80] | 39.00[36.50,41.40] | 39.40[37.40,41.80] | 39.30[37.20,41.90] | 39.50[37.00,41.80] | 0.218 |
| BUN, mmol/L | 6.36[5.28,7.58] | 6.29[5.13,7.46] | 6.25[5.18,7.35] | 6.38[5.23,7.63] | 6.46[5.50,7.85] | 0.11 |
| Scr, umol/L | 64.00[53.00,76.00] | 60.00[49.00,72.00] | 63.00[54.00,74.00] | 66.00[54.00,77.00] | 67.00[53.00,78.00] | 0.039 |
| FBG, mmol/L | 5.64[4.90,7.02] | 4.86[4.35,5.26] | 5.25[4.77,5.89] | 5.93[5.27,6.97] | 8.14[6.64,10.23] | <0.001 |
| UA, umol/L | 319.00[264.00,376.00] | 292.00[245.00,347.00] | 322.00[264.00,382.00] | 334.00[274.00,383.00] | 327.00[271.00,395.00] | <0.001 |
| TC, mmol/L | 3.48[2.92,4.07] | 2.96[2.65,3.51] | 3.53[3.02,4.00] | 3.61[3.14,4.15] | 3.83[3.23,4.49] | <0.001 |
| TG, mmol/L | 1.08[0.80,1.54] | 0.79[0.60,1.01] | 1.04[0.82,1.44] | 1.28[0.99,1.84] | 1.31[0.96,1.84] | <0.001 |
| HDL-C, mmol/L | 0.99[0.85,1.15] | 1.12[0.96,1.28] | 1.02[0.92,1.17] | 0.94[0.82,1.06] | 0.90[0.78,1.06] | <0.001 |
| LDL-C, mmol/L | 1.95[1.41,2.49] | 1.40[1.12,1.75] | 2.03[1.51,2.37] | 2.10[1.59,2.59] | 2.29[1.75,2.87] | <0.001 |
| NT-proBNP, pg/mL | 431.40[140.10,938.60] | 457.00[179.00,1043.00] | 392.10[121.00,800.60] | 442.600[130.00,938.60] | 435.00[104.70,925.10] | 0.297 |
| cTnT, ng/mL | 0.007[0.005,0.011] | 0.007[0.005,0.010] | 0.007[0.005,0.010] | 0.008[0.005,0.012] | 0.008[0.006,0.012] | 0.116 |
| LVEDD, mm | 48.00[46.00,52.00] | 49.00[46.00,52.00] | 48.00[46.00,52.00] | 48.00[46.00,52.00] | 48.00[46.00,51.00] | 0.29 |
| LVESD, mm | 31.00[28.00,34.00] | 30.00[28.00,33.00] | 31.00[28.00,35.00] | 31.00[28.00,35.00] | 31.00[28.00,34.00] | 0.165 |
| IVSD, mm | 8.00[8.00,9.00] | 8.00[8.00,9.00] | 8.00[8.00,9.00] | 8.00[8.00,9.00] | 8.00[8.00,9.00] | 0.73 |
| RVID, mm | 16.00[16.00,18.00] | 16.00[15.00,18.00] | 17.000[16.00,18.00] | 17.00[16.00,18.00] | 16.00[16.00,18.00] | 0.187 |
| LVEF, % | 67.00[62.00,70.00] | 67.00[63.00,70.00] | 66.00[61.00,70.00] | 66.00[61.00,70.00] | 66.00[62.00,69.00] | 0.34 |
| LAD, mm | 36.00[32.00,40.00] | 36.00[32.00,39.00] | 36.00[31.00,40.00] | 36.00[32.00,39.00] | 36.000[31.000,40.00] | 0.933 |
| CHG index | 5.22[4.96,5.48] | 4.76[4.59,4.87] | 5.10[5.04,5.15] | 5.33[5.26,5.41] | 5.70[5.58,6.00] | <0.001 |

AF, atrial fibrillation; ACEI/ARB, angiotensin-converting enzyme inhibitors / angiotensin II receptor antagonists; ALB, serum albumin; BMI, body mass index; BUN, blood urea nitrogen; CHD, coronary heart disease; CHG, cholesterol, high-density lipoprotein and glucose index; cTnT, cardiac troponin T; DBP, diastolic blood pressure; FBG, fasting blood glucose; HDL-C, high-density lipoprotein cholesterol; HR, heart rate; hs-CRP, high-sensitivity C-reactive protein; IVSD, interventricular septal depth; LAD, left atrial diameter; LDL-C, low-density lipoprotein cholesterol; LVEDD, left ventricular end-diastolic diameter; LVEF, left ventricular ejection fraction; LVESD, left ventricular end-systolic diameter; NT-proBNP, N-terminal pro-brain natriuretic peptide; OADs, oral antidiabetic agents; PLT, platelet; RBC, red blood cells; RVID, right ventricular internal diameter; SBP, systolic blood pressure; Scr, serum creatinine; TC, total cholesterol; TG, triglyceride; TyG, triglyceride glucose index; WBC, white blood cells.

**Table S2 ROC analysis results and DeLong test comparison results of AUC of the two models**

|  | **CHG index** | **TyG index** |
| --- | --- | --- |
| **AUC** | 0.765 | 0.673 |
| **Sensitivity** | 0.817 | 0.782 |
| **Specificity** | 0.581 | 0.504 |
| **Cut-off value** | 5.23 | 8.48 |
| **DeLong test P value** |  | <0.001 |

AUC, area under the curve; CHG, cholesterol, high-density lipoprotein and glucose index; ROC, receiver operating characteristic; TyG, triglyceride glucose index.

**Table S3 Discrimination of the baseline clinical model and the CHG-extended model for 12-month post-ablation atrial fibrillation recurrence.**

| **Model** | **Apparent C‑index** | **Bootstrap‑corrected**  **C‑index** | **Optimism** |
| --- | --- | --- | --- |
| Basic model | 0.628 | 0.600 | 0.028 |
| Basic model+ CHG | 0.753 | 0.735 | 0.017 |

CHG, cholesterol–HDL–glucose index. The basic model included age, sex, smoking and drinking status, diabetes mellitus, resting heart rate, body mass index, left atrial diameter, CHA₂DS₂-VASc score, and AF type.

**Table S4 Head-to-head NRI/IDI comparisons.**

| **Model 1** | **Model 2** | **NRI（95%CI）** | ***P*** | **IDI（95%CI）** | ***P*** |
| --- | --- | --- | --- | --- | --- |
| Basic model | Basic model+ FBG | 0.336 (0.140, 0.542) | 0.01 | 0.082 (0.029, 0.125) | <0.001 |
| Basic model | Basic model+ HDL-C | -0.022(-0.194, 0.244) | 0.174 | 0.017(-0.005, 0.068) | 0.279 |
| Basic model | Basic model+ TC/HDL-C | 0.264(0.039, 0.375) | 0.030 | 0.057(0.019, 0.102) | <0.001 |
| Basic model+ FBG | Basic model+ CHG | 0.241 (0.026, 0.441) | 0.030 | 0.118 (0.060, 0.198) | <0.001 |
| Basic model+ HDL-C | Basic model+ CHG | 0.507 (0.181, 0.665) | <0.001 | 0.183 (0.105, 0.235) | <0.001 |
| Basic model+ TC/HDL-C | Basic model+ CHG | 0.441 (0.207, 0.648) | <0.001 | 0.143 (0.079, 0.193) | <0.001 |

Basic model was adjusted for age, sex, smoking and drinking status, diabetes mellitus, heart rate, body mass index, left atrial diameter, CHA₂DS₂-VASc score and atrial fibrillation type. CHG, cholesterol-high-density lipoprotein-glucose index; CI, confidence interval; FBG, fasting blood glucose; HDL-C, high-density lipoprotein cholesterol; IDI, integrated discrimination improvement; NRI, net reclassification improvement; TC, total cholesterol.

**Figure S1 Receiver Operating Characteristic (ROC) curves for identifying 1-year atrial fibrillation recurrence using cholesterol-high-density lipoprotein-glucose (CHG) index and triglyceride-glucose (TyG) index. AUC: area under the curve.**

**Figure S2 Bootstrap-corrected calibration curves for predicting 12-month post-ablation atrial fibrillation recurrence.**

Bootstrap bias correction was performed with 1000 resamples to adjust for overfitting. The baseline clinical model included age, sex, smoking and drinking status, diabetes mellitus, resting heart rate, body mass index, left atrial diameter, CHA₂DS₂-VASc score, and AF type.

**Figure S3 Subgroup analysis for the associations between cholesterol-high-density lipoprotein-glucose (CHG) index and the incidence of 1-year atrial fibrillation recurrence. AF, atrial fibrillation; CI, confidence interval; DM,diabetes mellitus; HR, hazard ratio.**
